# A Call to Honesty: Extending Religious Priming of Moral Behavior to Middle Eastern Muslims

**DOI:** 10.1371/journal.pone.0099447

**Published:** 2014-07-03

**Authors:** Mark E. Aveyard

**Affiliations:** Department of International Studies, American University of Sharjah, Sharjah, United Arab Emirates; University of Pennsylvania, United States of America

## Abstract

Two experiments with Middle Eastern participants explored the generalizability of prior research on religious priming and moral behavior to a novel cultural and religious context. Participants in Experiment 1 completed a sentence unscrambling task with religious or non-religious content (in Arabic) before taking an unsupervised math test on which cheating was possible and incentivized. No difference in honesty rates emerged between the two groups, failing to extend findings from previous research with similar stimuli. Experiment 2 tested the effects of the *athan*, the Islamic call to prayer, using the same design. This naturalistic religious prime produced higher rates of honesty (68%) compared to controls who did not hear the call to prayer (53%).These results raise the possibility that the psychological mechanisms used by religion to influence moral behavior might differ between religions and cultures, highlighting an avenue of exploration for future research. The experiments here also address two growing concerns in psychological science: that the absence of replications casts doubt on the reliability of original research findings, and that the Westernized state of psychological science casts doubt on the generalizability of such work.

## Introduction

Numerous studies have demonstrated that when people process religious stimuli before an experimental activity their behavior changes under the influence of such stimuli (see [1] for an example involving volunteering). In the case of moral behavior in particular, findings generally show that people behave more morally under this influence (for a succinct review on prosociality effects, see Norenzayan & Shariff [2]; but for a counter-example, see Bushman, Ridge, Das, Key, & Busath [3]). In some cases, these effects emerge only for religious believers [4] and in other cases for non-believers also [5]. Cheating in performance situations (such as exams) often emerges as a measurement of ethical behavior in such experiments, due in part to the university-based context of most laboratory research. In one interesting study for example, participants who unscrambled sentences that included religious words like *cross* and *salvation* subsequently cheated less on a difficult task [5]. Given that correlational research over many years has shown little association between religiosity and cheating behavior [6], [7], these findings are somewhat surprising and, if reliable and generalizable, indicate that religious stimuli can prime ethical behavior regardless of participants' religiosity. One theory to explain these results involves the hypothesis that, in religions with omniscient gods, religious priming reminds believers that God is watching their actions (see Norenzayan [8] for a discussion of this supernatural monitoring hypothesis).

### Extending Religious Priming to Middle Eastern Muslims

But are such findings reliable and generalizable? Recent work in psychology has brought such “social priming” experiments under scrutiny due to numerous failures to replicate highly-cited, seminal experiments (for an example, see Harris, Coburn, Rohrer, & Pashler [9]; for empirical data showing low replication rates in psychology, see Makel, Plucker, & Hegarty [10]). The specter of the file drawer problem has also emerged with the recognition that traditional publishing practices might be suppressing statistically non-significant findings, thereby leading to inaccurate perceptions of psychological processes (for an analysis with statistical approaches for detecting publication biases, see Francis [11]). Likewise, concerns over the Westernized character of psychological science have led to calls for a more inclusive globalized program of psychological research [12], [13].

The experiments here aim to test prior religious priming findings in regard to cheating behavior. Advancing our theories of religiosity at the level of social and cognitive psychology remains the central task for researchers in this area, but it would be useful to know whether religious priming actually works as advertised when subjected to repeated attempts at replication, particularly outside Western settings. The practical significance of religious cognition in everyday life, particularly in regard to moral behavior, justifies some investment in replications. A growing empirical literature on religion and morality also compels us to explore the boundaries of previously published effects (for theoretical background, see Graham & Haidt [14]; for a literature review, see Preston, Ritter, & Hernandez [15]).

There are also features of prior studies that should limit our confidence in the generalizability of their findings. Very few studies involve Muslim majorities in their research samples (for an exception, see Bloom & Arikan [16] with Turkish participants), and to my knowledge, no prior experiments in religious priming have involved the participation of Muslims in Middle East, where Islam exerts a widespread influence on public and private life. (Nearly all prior work involves participant majorities who self-identify as Christians or live in cultures with Judeo-Christian norms.) Another limitation of prior research involves language: such experiments always use stimuli in English. Given known interactions in cognition between linguistic and conceptual processes (for a balanced discussion and review, see Wolff & Holmes [17]), constructing stimuli for different language groups offers a stronger replication test than conducting exact replications of previous research in English. The measured variable in the two experiments reported here constitutes a computer-based math test with a “programming error” that allows participants to improve their accuracy by cheating. Although this variable differs from those used in previous experiments on religious priming and honesty, it is conceptually consistent with those variables: Mazar, Amir, and Ariely [18] for example, used self-reported score on an incentivized performance task as a test of cheating. Furthermore, Shariff and Norenzayan [19] used the computer-error task in a correlational study on religious beliefs and cheating.

In summary, Experiment 1 differs from prior research in the due to cultural, religious, and linguistic factors (along with a new combination of independent and dependent variables). Should this experiment be characterized as a replication of previous work? Presently there is no consensus in psychology for categorizing studies along the dimension of novelty-and-replication. Kantowitz, Roediger, and Elmes [20] argue that replications can be sorted into three categories: direct replications (with very few changes in original methods), systematic replications (in which hypothetically irrelevant variables are manipulated to test the robustness of the original findings), and conceptual replications (which involve “radically different” methods than the original work). Nussbaum [21] offers a more expansive (and probably more common) definition of conceptual replication as a test of the “underlying hypothesis” of prior research “using different methods”. Although the experiments reported here seem to fall within the systematic replication category for Kantowitz et al [20] or the common definition of conceptual replications, such definitions offered by seem to regard replications conducted in similar cultural and linguistic contexts. Given the power of culture and language to shape our thoughts and behavior, extending a previously published method to broadly different cultural and linguistic contexts seems to go beyond the normal activity of replication, which usually occurs within the same culture, using the same language, often by collaborative research teams with similar participant populations. To avoid this terminological uncertainty, then, I use the term *extension* (rather than replication) as a descriptor for the experiments reported here.

## Experiment 1

Experiment 1 measured participants' honesty rates while taking a mathematics test after unscrambling religious or non-religious sentences in Arabic. The scrambled sentence task represents a common kind of stimuli used in previous religious priming studies (Randolph-Seng & Nielsen [5]; Shariff & Norenzayaan [22]). Participants see five words which can be used to form a sensible four-word sentence. The task requires discarding one word that does not fit grammatically or semantically with the other words.

### Ethics Statement

The rights of our participants were protected according to the guidelines of Institutional Review Board at the American University of Sharjah, which approved this research. Participants provided signed consent after reading a description of the procedures.

### Methods

Participants were 70 Arabic-speaking Muslim undergraduates (46 females, 24 males) at a university in the United Arab Emirates participating for course credit. The first phase of the experiment required participants to unscramble a set of five words in Arabic to make a sensible sentence, leaving out one word that did not belong. Participants were randomly assigned to one of two conditions: the experimental condition, in which 5 of 10 sentences had religious content, and the control condition, which included the 5 non-religious sentences from the experimental condition and 5 additional non-religious sentences (see Appendix S1). These Arabic sentences were modeled on the English sentences used by Shariff and Norenzayaan [22] in a study of religious priming and prosocial behavior (and subsequently by Toburen and Meier [23] in a study of religious priming and task persistence).

The experimenter presented the study as an exploration of the relationship between language and mathematical abilities. After explaining the scrambled sentence task, participants were also introduced to the second phase of the procedure: a time-limited test of math skills in which they would add arrays of numbers to produce a sum (e.g., “−10 −13 −6 −3 −10 16 −9 0 8” which yields “−27”) under time pressure (15 seconds per problem). To induce cheating behavior, this phase used a modified version of the “computer-glitch” procedure from von Hippel, Lakin, and Shakarchi [24] with a critical ploy: the answer appears on screen unless participants press a key to prevent it from appearing. Three practice items were presented to confirm that participants understood the instructions. After allowing the answer to appear for the first practice item (by pretending to be occupied with paperwork), the experimenter stated that the answer appeared on screen due to an error in the computer program that had not yet been fixed. Participants were told that they could prevent the answer from appearing by pressing any key, such as the spacebar, before the answer box appeared (there was a 7 second window between the problem's disappearance and the answer's appearance for participants to press a key).

The experimenter then concluded the instructions with several statements: that he or she would be down the hallway helping other participants during the study; that the program would terminate as soon as a 70 percent accuracy threshold was reached; that participants would be finished with the study at that point; that they would receive full credit for finishing the study regardless of the number of problems they completed; and that they should gather their belongings and find the experimenter in the hallway afterwards for debriefing and credit assignment. The experimenter then informed participants that their overall accuracy rate would appear on screen after 10 problems and that the new problems would continue to appear until that rate exceeded 70 percent (in reality, if this figure was not reached after 70 trials, the program automatically terminated). The experimenter then left the room, closed the door, and each participant completed the experiment alone, starting with the sentence unscrambling task. Since nearly all of our participants regard such repetitive arithmetic work as unpleasant and difficult, the incentive to cheat involved reaching the end of the experiment more quickly. The experiment used E-Prime experimental software.

### Results

Of 70 participants, 5 did not press the space bar at all during the experiment, indicating that they might have misunderstood the math task instructions or forgotten them during the intervening sentence unscrambling task. Seven other arithmetically-skilled participants were removed who scored above 70 percent on trials in which they prevented the answer from appearing: their algebraic skills rendered cheating unnecessary to exceed the 70 percent accuracy threshold.

**Figure 1:**
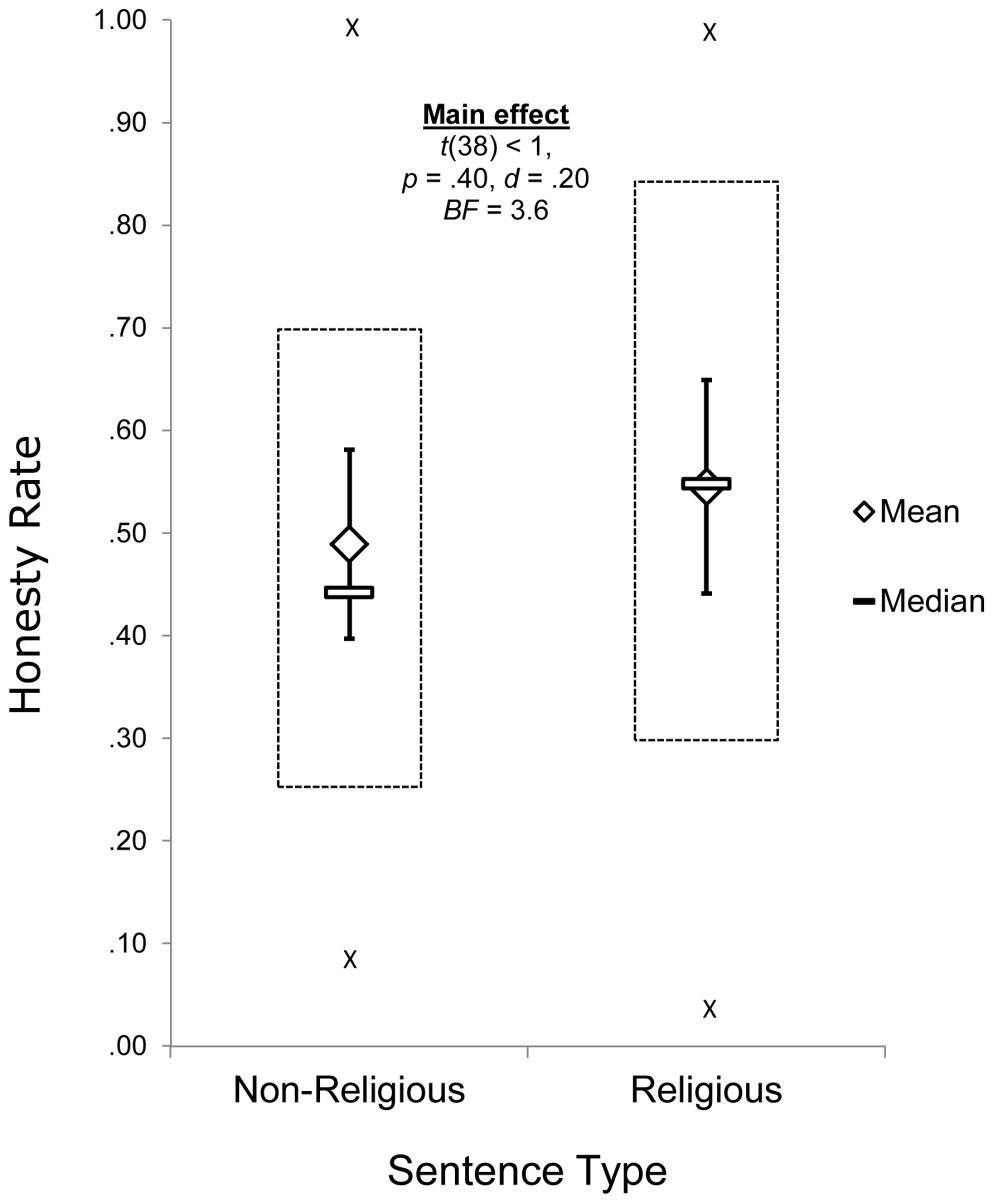
Honesty rates for participants who unscrambled religious(Call) and controls. Vertical bars: 95% confidence intervals. Boxes: 1st and 3rd quartiles. “X”: max and min values.

Of the remaining participants, those who were exposed to sentences with religious content showed honesty rates (*N* = 35, *M* = .49, *SD* = .28) that were not significantly different than participants in the control condition (*N* = 33, *M* = .55, *SD* = .30), *Means Difference (MD)* = .06, *MD SE* = .07, *MD* 95% CI [−.19, .08], *t*(66) <1, *p* = .40, *Bayes Factor (BF)* = 3.6, 95% CIs for conditional means [Experimental: .66, .34; Control: .72, .43] (Figure 1). Unlike some experiments using the sentence unscrambling task, Experiment 1 here did not produce a difference in cheating behavior between the religious and non-religious priming conditions. The observed pattern trended slightly in the opposite direction of previous research using the same religious priming techniques. Although the sample size for this study (*N* = 68) was large enough to detect only a strong effect of the manipulation, the religiosity condition actually showed nominally lower honesty rates compared to the control condition. Thus, even a modest pro-honesty effect of the manipulation would require many more participants to emerge, on the order of hundreds. Such a sample size would far exceed the sample sizes used in previously published studies.

## Experiment 2

Experiment 1 failed to extend priming effects seen in previous research using scrambled sentences with religious content. Given the Western setting of most religious research, it is tempting to hypothesize that unidentified cultural and religious factors might mediate the influence of religious priming. But the range of possible mediators would be too large to explore in a limited program of research, as they could span an array of variables from theological content to language differences to cultural norms. In our laboratory discussions, though, increasing the emotional valence of religious stimuli emerged as a possible technique that could induce higher rates of honesty. Previous studies include a number of other priming techniques besides the scrambled sentence task inspired by decades of research in cognitive psychology: lexical decision tasks [25], word search puzzles [1], and subliminal primes preceding lexical decisions [26], [27]. But these simple verbal stimuli carry very little emotional relevance compared to the symbols, rituals, and materials that animate daily religious beliefs and practices, especially among Muslims in the Middle East (for a study exploring ecological validity and religious priming, see LaBouff [28]).

Indeed research on other topics shows that different types of experimental primes can produce different behavioral effects. Some studies have found that the personal relevance and affective valence of stimuli, for example, can change the effectiveness of primes (regarding stimuli relevant to patients with chronic pain, see Dear, Sharpe, Nicholas, & Refshauge [29]; regarding affect and individual differences, see Zemack-Rugar, Bettman, & Fitzsimons [30]). Given the results of Experiment 1, there is reason to suspect that the specific characteristics of religious primes could matter more than previous studies might indicate. Naturalistic primes in particular might influence moral behavior where artificial primes fail to do so. In daily life, naturalistic stimuli can be used effectively for religious purposes: a cross worn around the neck, for example, can serve as a personal admonition to think and behave morally. At least one study on religious priming has involved naturalistic stimuli: researchers asked a subset of participants to recall the Ten Commandments (from the Jewish Torah or the Christian Old Testament) and observed a subsequent reduction in cheating behavior for those participants compared to controls [18]. Such religious verses, though naturalistic, do not represent the kind of religious symbols or rituals that impact believers on a daily basis (for most believers). And so far researchers have not tried to contrast these effects with the artificial primes normally used in these studies.

In Islam the *athan*, or call to prayer, serves as a public reminder of believers to fulfill their religious obligations and carries a stronger emotional valence than scrambled sentence stimuli from Experiment 1. Using the same procedures and measurements otherwise, Experiment 2 replaced the scrambled sentence task with this naturalistic stimulus: an audio file of city traffic with the call to prayer in the background or the same audio file without the call to prayer.

### Methods

Participants were 88 Arabic-speaking Muslim undergraduates (56 females, 23 males) in the same location as Experiment 1. Non-Muslims (*N* = 13) and non-Arabic speaking Muslims (*N* = 16) were allowed to participate in Experiment 2 for course credit. To maintain consistency in sample characteristics across Experiments 1 and 2, their data was not analyzed. In any case, the small sample sizes for each group would render such analysis entirely inconclusive. The first phase of the experiment required participants to listen to a two-minute audio recording of traffic, with various sounds typical of a busy city street. Participants were randomly assigned to one of two conditions: the experimental condition, in the call to prayer appears 45 seconds into the 2 minute audio file (and continues in the background until the end of the file), and the control condition in which the call to prayer was not included. The experimenter presented the study as an exploration of the relationship between driving conditions and mathematical abilities. Participants were asked to count the number of horns that sounded from vehicles during the audio file and enter their estimate in a box on screen. The experimenter also explained the second phase of the experiment involving the same math task used in Experiment 1. This experiment also used E-Prime software. Raw data files for both experiments are publically accessible at Open Science Framework (https://osf.io/ah69g/).

### Results

**Figure 2:**
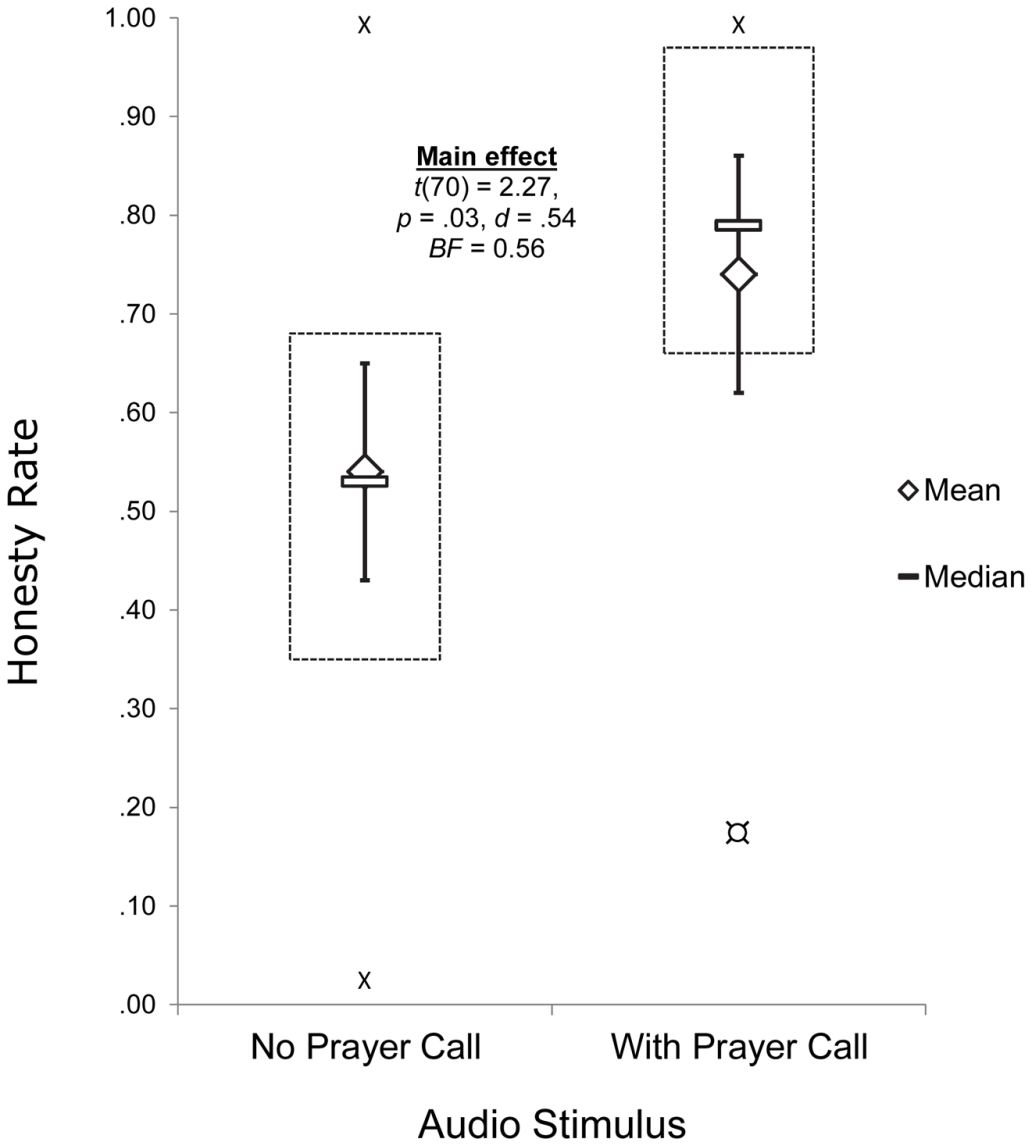
Honesty rates for participants exposed to the call to prayer and not exposed. Vertical bars: 95% confidence intervals. Boxes: 1st and 3rd quartiles. “X”: max and min values. “O”: outlier (2 *SDs*).

Of 88 participants, 9 participants in the post-experiment interview reported suspicion about the experiment's purpose during the procedure; 3 did not press the space bar at all during the experiment, indicating that they might have misunderstood the instructions or forgotten them due to the intervening listening task; and 4 other participants were removed who scored above 70 percent on honest response trials (rendering cheating unnecessary toward reaching the required 70 percent threshold). No participants in the experimental condition expressed suspicion in regard to religiosity. Of the remaining 72 participants, those exposed briefly to the call to prayer showed a higher honesty rate (*N* = 36, *M* = .68, *SD* = .29) compared to participants in the control condition (*N* = 36, *M* = .53, *SD* = .27), *MD* = .15 *MD SEM* = .07, 95% CI [.28, .02], *t*(70) = 2.27, *p* = .03, *d* = 0.54, *η^2^* = .07, *BF* = 0.56, 95% CIs for conditional means 95% [Experimental: .78, .58; Control: .62, .44] (Figure 2) . D'Agostino-Pearson normality testing showed normal distributions in the experimental condition (*K2* = 5.76, p = .06) and the control condition (*K2* = 1.34, p = .38). It could be argued that the borderline abnormality in the experimental condition (confirmed by visual analysis) calls for a nonparametric analysis. A Mann-Whitney test produced similar results as the parametric analysis (Experimental *Mdn* = .78, Control *Mdn* = .50), *Z* = 2.16, *p* = .03. Interestingly, participants showed nearly the same overall honesty rate in the control condition (53%) as controls in Vohs and Schooler [31] with a shorter version of the same task (51%) as well as controls in the original computer glitch study by von Hippel et al [24] in the comparable condition (51%). In a correlational design, Shariff and Norenzayan [19] used the same task to compare participants' God concepts (whether they view God as punishing or forgiving) with their cheating rates. In this study the average cheating rate across participants was 55%, again showing similar rates to controls here. The larger sample size for Experiment 2 compared to Experiment 1 was an artifact of additional participants requesting participation credit for their courses. Analyzing the data chronologically, *p* moves below and above .05 throughout data collection. As such, no particular significance should be attached to the reported value here falling below .05, as a much larger sample size would be required to reliably achieve lower error probabilities. In this case, resource limitations led to the termination of the experiment.

The math task involving the cheating measurement lasted for 24 minutes on average after exposure to the audio file. The temporal strength of the main effect can be measured by marking the last trial during which participants pressed the space bar compared to the total number of trials, yielding an honesty-endurance ratio for each participant (e.g., .90 for a participant with 40 trials whose last space bar press occurred on trial 36). Participants in the experimental condition showed a longer duration of honesty (*Mdn* = .99) compared to control participants (*Mdn* = .91), *Z* = 2.17, *p* = .03. Normality testing indicated non-normal distributions in the experimental condition (K2 = 15.28, p<.001) and control condition (K2 = 14.39, p<.001). Ceiling effects make it difficult here to test the difference between medians, so median difference of .08 is possibly more significant than it might appear.

A post-experiment interview tested manipulation awareness by asking participants what else they heard during the audio file besides the horns, what they were thinking about while completing the math task, and what they thought was the purpose of the study. Follow-up questions asked them to assess how often they failed to press the space bar and why. In the experimental condition, no participants made a connection between the call to prayer and cheating on the math task at any point during the interview. Most participants (84%) reported that intentionally failing to press the space bar constituted an act of cheating and most participants (86%) in the experimental condition reported hearing the call to prayer among other background noises. Across both conditions, six participants came to suspect that the experiment was really about measuring cheating behavior but this suspicion arose only during the interview and not during the task itself (as opposed to the nine participants who reported such thoughts as occurring during the study procedures). As such, the effects reported here, while involving conscious processing (of both the call to prayer and the opportunity for cheating) cannot be attributed entirely to participants actively making a connection between the manipulation and the measurement.

Several weeks before the experiment, participants completed a prescreen questionnaire that included four questions related to religion and sociality: specifically, the level of self-reported religiosity, spirituality, belief in a universal design, and general concern for others. None of these variables (in addition to sex) moderated the main effect in a linear regression analysis, although the sample size (*N* = 72) was possibly too small to detect moderating relationships. Participants reporting lower general religiosity (0 to 4 on the 7-point scale, *N* = 39) showed little or no increase in honesty levels (61% in the experimental condition, *N* = 20, to 59% in the control condition, *N* = 19) compared to participants reporting higher levels of general religiosity (5 to 7 on the 7-point scale, *N* = 33, 77% in the experimental condition, *N* = 16, to 46% in the control condition, *N* = 17). At first glance it would seem that general religiosity should have moderated the main effect. But the 11 participants who rated themselves lowest on the religiosity scale (from 0 to 2) also showed higher increases in honesty rates (81% in the experimental condition, *N* = 6, to 61% in the control condition, *N* = 5). In other words, participants who reported religiosity in the middle of the scale seemed uninfluenced by the manipulation. Thus a linear analysis fails to reveal a moderating influence of this variable. Some studies in religious priming report a moderating role for self-reported religiosity [5] while others report no moderating role [18]. So, in addition to the sample size of Experiment 2, a nonlinear analysis is limited by the absence of a clear theoretical reason to believe that those differences reflect something other than random sampling fluctuations.

## Discussion

Two attempts to extend religious priming research produced mixed results in a previously unexplored population of participants, Middle Eastern Muslims. While listening to an audio file featuring the sounds of a busy city street, participants in Experiment 2 counted the number of horns they heard sounding from vehicles. In the experimental condition, the Islamic call to prayer played in the background starting 45 seconds into the 2 minute audio file. On a subsequent math task participants exposed to this condition cheated significantly less often than participants in the control condition, who completed the same audio task without the call to prayer in the background. The math task involving the cheating measurement lasted for 24 minutes on average after exposure to the audio file. Under these conditions, the results show a remarkably strong and enduring influence of religious priming on participant behavior. Also, self-reported religiosity (“How religious are you?” on a 7-point scale, from a prescreen questionnaire several weeks earlier) did not moderate the main effect in a linear way.

Experiment 1, however, failed to extend previous research using similar sets of artificial stimuli [19], [22]. Why did Experiment 2 show effects similar to other studies while Experiment 1 did not? Given the sample sizes in both experiments, it is possible the observed data produced statistical values that differ significantly from the true means of the sampling distributions. Otherwise, at least two interpretations are worth considering: first, the frequency of exposure to religious stimuli in daily life might affect the threshold of activation for religious cognition in a laboratory setting; and second, regardless of such exposure, some religious systems might use different mechanisms to activate morally-based self-control. In regard to the first cognitive-threshold explanation, we would need to reconcile Western findings with the results of Experiment 1 here. Assuming the published research cited above is not an artifact of the file drawer effect and related problems in research practices, it would be worth noting that religious priming effects in prior research emerged from Western settings that have been become widely secularized. Possibly nearly any type of religious stimuli could activate systems of religious cognition beyond the threshold required to influence subsequent decision-making. Experiencing such stimuli in a Western university setting (often associated with non-religious or even anti-religious mindsets) might lower the threshold of activation even more. Regarding the idea that people behave more ethically when their belief in an omniscient God is activated, Gervais and Norenzayan [32] demonstrated that religious priming, via a sentence unscrambling task, can increase public self-awareness, and that the same task can elicit higher levels of socially-desirable responding among people who report higher levels of God belief. Given the systematic theologies present across monotheistic traditions, which include clear rule-based moral prescriptions, it is plausible to assume that the same mechanisms will work among believers across these traditions. But Experiment 1 here failed to produce evidence of a supernatural monitoring effect using the same priming method from previous studies. Possibly then, supernatural monitoring can be effective only with substantial above-normal activation of religious cognition, which was achieved in this population with the call to prayer in Experiment 2. For Muslims in the Middle East, simple religious sentences might not be strong enough to activate religious cognition, as both public and private life is more widely filled with religious verbiage. Common verbal rituals (like salutations and valedictions) involve religious words. It is possible that presenting verbal religious stimuli does not provide sufficiently strong information to affect subsequent behavior for these kinds of participants, at least on tasks that feature ethical dilemmas. So a particularly strong religious signal could be necessary, such as the call to prayer. Although the call to prayer is also a daily feature of life in Muslim regions, it is encountered less frequently than religious verbal stimuli and it evokes stronger behavioral demands for Muslims. It is possible that non-Muslims who live in Muslim societies would show also show higher honesty rates after exposure to the call to prayer. Neither the cognitive-threshold explanation above nor the affective response explanation requires that a person self-identify with Islam to show these effects. Indeed, as noted above, a simple measure of self-reported religiosity among Muslims did not clearly moderate the effect. So perhaps the effect would emerge in non-Muslims with a large enough sample size. What is necessary, perhaps, is that the call to prayer merely be encoded as an important, affective symbol related to moral behavior.

Alternatively, perhaps Middle Eastern Islam makes use of affective processes through its symbols and rituals. Perhaps the effects in Experiment 2 emerged not because religious cognition failed to activate above normal thresholds but because the call to prayer automatically stimulates affect. Indeed the *athan* can become an emblem of political and ethnic controversies beyond its basic religious purpose. In Singapore, for example, a large resettlement project in the 1960s and 1970s included the relocation of some Malay Muslims to dense urban areas. As new mosques began to broadcast the amplified call to prayer over loudspeakers, non-Muslim populations began to contest what they perceived as an invasion of public space, a situation that was eventually resolved with radio broadcasting of the call to prayer [33]. The contentious nature of such symbolism and communication illustrates the emotional resonance of such stimuli compared to others. The affect-based explanation raises the possibility that religions or religious cultures could differ in the way that they use religion to encourage moral behavior. Some religious cultures might use more cognitive routes to moral behavior and others more affective routes. As such certain religious primes might serve as effective manipulations in some cultures and not in others, which make use of other behavioral mechanisms.

Most prior work on religious priming does not explore, in depth, the mechanisms by which religious stimuli might be achieving its effects (for recent exceptions, see Preston & Ritter [34], and Rounding, Lee, & Jacobson [35]; but see Harrison & McKay [36] as a failure to replicate the latter). The results here argue for a more complex exploration of these mechanisms, revealing that not all religious primes are created equal. In experiencing failures to replicate or extend previously published findings, researchers should consider using different types of religious stimuli to activate different mechanisms for influencing behavioral measurements. In this respect, God is in the details. These results also highlight the importance of achieving adequate sample sizes in original research studies with anticipation of future work that could replicate or extend the findings. The two experiments here aimed for sample sizes similar to prior research in this area (although Experiment 2, *N* = 72, represents a larger sample size than most previous studies). Yet even Experiment 2, which indicated an influence of religious priming, was somewhat underpowered (.73 achieved power, one-tailed) for the observed effect size. Given the outcome of Experiment 1 (a non-extension of prior work), it is possible that religious priming, when it works, is much weaker than previous research indicates. When attempting to extend prior work, researchers should not assume that published sample sizes offer sufficient estimates for planning research. Also, most prior research on this topic reports *p* values that are not strongly significant: Randolph-Seng & Nielsen [5] report values of .02 (twice; *N* = 45 and *N* = 49); Shariff and Norenzayan [19] report values of .04 (twice; *N* = 61 and *N* = 39); and Mazar, Amir, and Ariely [18] report a value of .02 (*N* = 229). These values indicate achieving sufficient power in an experiment will often require sample sizes that greatly exceed those reported here.

While Experiment 2 here showed a positive effect of the call to prayer, previous research indicates that religious primes can have negative influences on behavior also: in making people more aggressive, for example, after reading scriptural texts describing violence sanctioned by God [3]. Also, other research using the computer-glitch technique shows that people primed with a ghost story also show reductions in cheating behavior [37], which raises questions about other mediating factors. Additionally, the experimental task here involved cheating by omission (not pressing a button) instead of commission, a structure that might lead to increased levels of cheating, commonly referred to as *omission bias* (in line with original research by Spranca, Minsk, & Baron [38]; but see Baron & Ritov [39] and DeScioli, Bruening, & Kurzban [40] for the limiting contexts of this bias). Descioli, Christner, and Kurzban [41] have shown that omission bias might be particularly relevant when immoral actions can be punished by a third party. Finally, an ongoing debate about religious stimuli inducing the “imagined presence of supernatural watchers” [19] remains an unresolved yet pertinent issue about tasks used in religious priming experiments.

The experiments reported here follow a growing body of research showing various effects of religious primes on moral behavior. Few studies from independent laboratories have extended previous research on religious priming, especially outside Western settings. Additionally, previous experiments have shared several features: they use artificial religious primes (rather than stimuli with personal significance), Judeo-Christian stimuli, and the English language. When artificial religious primes in Arabic failed to affect honesty rates in Experiment 1 among Middle Eastern Muslim participants, Experiment 2 here used a daily feature of Islamic religious practice, the call to prayer, to elicit behavioral changes.

## Supporting Information

Appendix S1 Scrambled sentence stimuli for Experiment 1.
(DOCX)
