# Supplementary material for: A Call to Honesty: Extending Religious Priming of Moral Behavior to Middle Eastern Muslims
Source: PLoS One. 2014 Jul 3;9(7):e99447. doi: 10.1371/journal.pone.0099447 (PMC4084630; doi:10.1371/journal.pone.0099447)
Supplement: Appendix S1 — Scrambled sentence stimuli for Experiment 1. (DOCX) [file pone.0099447.s001.docx]

**Appendix**

**Sentence Stimuli (Scrambled) for Experiment 1**

**Religious Condition**

الولد الكتاب المدرسة إلى ذهب

يوم غرفتها المدرسة ترتب كل

الرجل ذهب الصلاة يلعب إلى

الأطفال يشرب الشاي كل يوم الحليب

المسلمون يذهب سنويًا يوميًا للحج

الطفل أمي الحديقة يلعب في

الفتاة صامت لقد غدُا البارحة

الولد كتابًا لعبة اشترى جديدًا

صالحُا ننام الجنة نعمل لندخل

نذهب الله دائمًا معنا إن

**Non-religious Condition**

الولد الكتاب المدرسة إلى ذهب

يوم غرفتها المدرسة ترتب كل

الولد الحديقة تلفاز ذهب الى

الأطفال يشرب الشاي كل يوم الحليب

الرياضة البيت للجسم ممارسة مفيدة

الطفل أمي الحديقة يلعب في

رضا فاشل تمساح شخص بالدراسة

الولد كتابًا لعبة اشترى جديدًا

مباني جدا الحاسوب الجامعة ضخمة

هذا الرجل كالماء كالأسد ق
